# The Effect of Physical Therapy Treatment in Patients with Subjective Tinnitus: A Systematic Review

**DOI:** 10.3389/fnins.2016.00545

**Published:** 2016-11-29

**Authors:** Sarah Michiels, Sebastiaan Naessens, Paul Van de Heyning, Marc Braem, Corine M. Visscher, Annick Gilles, Willem De Hertogh

**Affiliations:** ^1^Department of Rehabilitation Sciences and Physiotherapy, Faculty of Medicine and Health Sciences, University of AntwerpAntwerp, Belgium; ^2^Department of Otorhinolaryngology, Antwerp University HospitalEdegem, Belgium; ^3^Multidisciplinary Motor Centre Antwerp, University of AntwerpAntwerp, Belgium; ^4^Department of Translational Neurosciences, Faculty of Medicine and Health Sciences, University of AntwerpAntwerp, Belgium; ^5^Department of Special Care Dentistry, Antwerp University HospitalEdegem, Belgium; ^6^Department of Oral Health Sciences, Academic Centre for Dentistry Amsterdam, University of Amsterdam and VU University Amsterdam, Research Institute MOVE AmsterdamNetherlands; ^7^Department of Social Welfare, University College GhentGhent, Belgium

**Keywords:** somatic tinnitus, physical therapy, treatment, cervical spine, temporomandibular joint disorders

## Abstract

**Background:** Tinnitus is a very common symptom that often causes distress and decreases the patient's quality of life. Apart from the well-known causes, tinnitus can in some cases be elicited by dysfunctions of the cervical spine or the temporomandibular joint (TMJ). To date however, it is unclear whether alleviation of these dysfunctions, by physical therapy treatment, also decreases the tinnitus complaints. Such physical therapy could be an interesting treatment option for patients that are now often left without treatment.

**Objectives:** The aim of this review was to investigate the current evidence regarding physical therapy treatment in patients with tinnitus.

**Data sources:** The online databases Pubmed, Web of Science, Cochrane, and Embase were searched up to March 2016. Two independent reviewers conducted the data extraction and methodological quality assessment.

**Study eligibility criteria:** Only randomized controlled trials and quasi-experimental trials were included in the review. Studies had to be written in English, French, Dutch, or German.

**Participants and interventions:** The included studies investigated the effect of physical therapy treatment modalities on tinnitus severity in patients suffering from subjective tinnitus.

**Results:** Six studies were included in this review, four investigating cervical spine treatment and two investigating TMJ treatment. These studies show positive effects of cervical spine treatment (manipulations, exercises, triggerpoint treatment) on tinnitus severity. Additionally, decrease in tinnitus severity and intensity was demonstrated after TMJ treatment, following splints, occlusal adjustments as well as jaw exercises.

**Limitations:** The risk of bias in the included studies was high, mainly due to lack of randomization, lack of blinding of subjects, therapists, and/or investigators. Additionally, risk of bias is present due to incomplete presentation of the data and selective reporting. A major issue of the reviewed papers is the heterogeneity of the included study populations, treatments and outcome measures, which inhibit data pooling and meta-analysis.

**Conclusions:** Despite the methodological issues in the included studies and the consequent low quality evidence, it is noteworthy that all included studies show positive treatment effects. Before recommendations can be made, these results need to be confirmed in larger, high quality studies, using unambiguous inclusion criteria, state-of-the-art treatment, and high quality outcome measures.

## Introduction

Tinnitus or “ringing in the ears” is a conscious perception of an auditory sensation in the absence of a corresponding external stimulus (Baguley et al., 2013). It is a very common symptom [15% of the adult population (Axelsson and Ringdahl, 1989)] that often causes distress and decreases the patient's quality of life. The ability to do intellectual work can be negatively affected and sleeping difficulties are frequently reported (Baguley et al., 2013).

Various types and causes of tinnitus have been described, with two main subtypes of tinnitus described: A subjective and an objective type. Tinnitus is in most cases subjective, meaning that the patient experiences the tinnitus in the absence of any auditory stimulus. In some cases, an internal, measurable, stimulus can cause the tinnitus, for instance turbulences of the blood flow. In these cases the perceived somatosounds can be considered an objective tinnitus (Baguley et al., 2013).

Several risk factors for subjective tinnitus have been described, such as hearing loss (Domenech et al., 1990), ototoxic medication (e.g., salicylates), head injuries (Ceranic et al., 1998), and depression (Trevis et al., 2016). Tinnitus can also occur in association with otological conditions, such as noise exposure or presbyacusis and can co-exist with anxiety or depression (McKenna et al., 1991) and with dysfunctions of the cervical spine (Teachey et al., 2012) or temporomandibular joint (TMJ) (Saldanha et al., 2012).

In these last two cases, tinnitus can be elicited by the somatosensory system of the cervical spine or temporomandibular area. This type of tinnitus is called somatic tinnitus and has been described in 36–43% of a population with subjective tinnitus (Abel and Levine, 2004; Michiels et al., 2015). A physiological explanation is proposed by several animal studies, which have found connections between the somatosensory system of the cervical spine and temporomandibular area on the one hand and the cochlear nuclei (CN) on the other hand (Pfaller and Arvidsson, 1988; Zhan et al., 2006). Cervical and temporomandibular somatosensory information is conveyed to the brain by afferent fibers, the cell bodies of which are located in the dorsal root ganglia or the trigeminal ganglion. Some of these afferent fibers also project to the central auditory system and more specifically to the dorsal CN. This makes the somatosensory system able to influence the auditory system by altering the spontaneous rates (i.e., not driven by auditory stimuli) or the synchrony of firing among neurons in the CN, inferior colliculus or auditory cortex. In this way, the somatosensory system is able to alter the intensity and the character of the tinnitus for instance by forceful muscle contractions of the neck or jaw musculature (Levine, 1999; Shore et al., 2007) or by increased muscle tension in the tensor tympani muscle (Westcott et al., 2013). Langguth et al. (2007) already stated that the investigation of the cervical spine and TMJ should be considered in all subjective tinnitus patients.

In 2011, Sanchez et al. (Sanchez and Rocha, 2011) published a literature overview on the diagnosis and treatment of somatic tinnitus, proposing the now currently used diagnostic criteria for somatic tinnitus. Regarding therapy however, this literature overview was not systematically performed and was based on case studies and case series that do not provide high quality evidence for the effect of physical therapy treatment in tinnitus patients.

Physical therapy could be an interesting treatment option for patients that are now often left without treatment. Therefore, the aim of this review was to investigate the current evidence regarding physical therapy treatment in patients with tinnitus.

## Methods

### Search strategy

A search of the online databases Pubmed, Web of Science, Cochrane, and Embase was performed up until March 2016. The search strategy was based on the PICO-framework and the following search was entered in the different databases: [(“Tinnitus”[Mesh]) AND (“Exercise Movement Techniques”[Mesh]) OR (“Musculoskeletal Manipulations”[Mesh]) OR (“Exercise Therapy”[Mesh]) OR (“Myofunctional Therapy”[Mesh])].

### Systematic review registration number

A detailed review protocol was composed by the authors and registered at PROSPERO (registration number: CRD42016035834).

### Study selection

For inclusion in the review, studies needed to meet the following inclusion criteria: (1) subjects had to be human, (2) patients had to be adults suffering from subjective tinnitus, (3) the studied intervention was a physical therapy treatment modality, (4) this treatment was compared to no treatment or another treatment, (5) a tinnitus severity measure was one of the outcome measures, (6) studies had to be written in English, French, Dutch, or German, (7) only randomized controlled trials and quasi-experimental trials were considered for inclusion and (8) articles had to present original research. Articles not meeting all inclusion criteria were excluded.

After the initial search, all retrieved articles were screened for eligibility based on title and abstract. The included articles were then screened again based on the full text.

The inclusion procedure was conducted by the first and third author independently and supervised by the last author. In case of uncertainty about inclusion, a decision was made in a consensus meeting, starting from the three independent opinions.

### Qualification of the investigators

The literature was screened and methodological quality was assessed independently by the first author, PhD. with experience in tinnitus and neck related complaints, and by the second author, MSc. in rehabilitation sciences and physiotherapy. The last author, PhD. with experience in neck related complaints, supervised the process. The third and sixth author, provided overall expertise on tinnitus complaints and the fourth and fifth author provided overall expertise on temporomandibular dysfunction.

### Data items and collection

All relevant information from each included article was extracted and is presented in Tables 1, 2. This table contains the number of patients, used outcome measure for tinnitus severity and the main findings.

**Table 1:** **Summary of studies concerning cervical spine treatment**.

**Publication**	**Participants**	**Intervention and control**	**Frequency and duration of intervention**	**Tinnitus severity outcome**	**Follow-up**	**Results**
Amanda et al., 2010	*N* = 40 Females: 10 Males: 30	Osteopathic manipulations of the cervical spine	Once a week for 2 months	Tinnitus Handicap Inventory (THI)	Post-treatment	**THI**: No difference between treatment groups: TENS: 15.1 points recuction (−27%; *p* <0.001) Manip: 8.5 points reduction (−16.2%; *p* <0.04)
	Age: 48.5 (18–65)	vs.		VAS-intensity		
	Design: RCT					**VAS-intensity**: No improvement manip., 1.45 point decrease (*p* <0.006) in TENS
	Diagnosis: Tinnitus patients otherwise healthy	Transcutaneous electrical nerve stimulation (TENS)				
Latifpour et al., 2009	*N* = 24 Females: 12 Males: 12 Age: 51 (*SD*: 16) Design: Controlled trial Diagnosis: Somatically related tinnitus	Supervised self-stretch of shoulder, neck and jaw muscles (Deltoid, trapezius pars descendens, splenius capitis, levator scapulae and sternocleido-mastoideus, masseter, temporalis and pterygoid), combined with Posture exercises and Auricular accupunture	9 sessions of 60 min, 3 per week during 3-week period	VAS-severity	Post-treatment and 3 months follow-up	**VAS-severity**: Significantly greater decrease in treatment group compared to controls after treatment (*p* = 0.001) and after follow-up (*p* = 0.006)
		vs.				
		Waiting list				
Mielczarek et al., 2013	*N* = 80 Females: 38	TENS	15 TENS applications in a period of 30 days	Author's own questionnaire	Post treatment, 1 and 3 months follow-up	Significant improvement in both groups
	Males: 42 Age: 21–74	vs.				
	Design: Controlled trial	Cervical physical therapy (stabilizing and mobilizing exercises)				No significant difference between groups
	Diagnosis: Tinnitus and sensorineural hearing loss + cervical spine degenerative changes (radiologically diagnosed)					
Rocha and Sanchez, 2012	*N* = 71 Females: / Males: / Age: /	Ischemic compression therapy of trigger points, stretching and posture exercises	10 weekly sessions	Tinnitus Handicap Inventory (THI)	Not mentioned	Improvement in THI in the fifth session (*p* <0.001)
	Design: RCT	vs.				
	Diagnosis: Tinnitus and pain complaints in head, neck or shoulder girdle during the previous 3 months	Sham deactivation trigger points				

**Table 2:** **Summary of studies concerning temporomandibular treatment**.

**Publication**	**Participants**	**Intervention and control**	**Frequency and duration of intervention**	**Tinnitus severity outcome**	**Follow-up**	**Results**
Tullberg and Ernberg, 2006	Patients (P): *N* = 73 Controls (C): *N* = 50 Females: 39 (P) / 27 (C) Males: 34 (P) / 23 (C) Age: 48 (*SD*:12) (P) 47 (*SD*:14) (C)	Splints, occlusal adjustments, jaw exercises and laser therapy vs. Waiting list	1 to 6 sessions	Global perceived effect (GPE) Custom made questionnaire	Post-treatment (GPE) and 2–3 years follow-up (questionnaire)	**GPE**: 73% reported improvement, 27% reported no change
						**Questionnaire**: Significanty decreased tinnitus severity Significantly more improvement in the patients than in the control group
	Design: Controlled design					
	Diagnosis:Patients suffering from combination of tinnitus and TMD Controls suffering from tinnitus					
Erlandsson et al., 1991	*N* = 32 Females: 14 Males: 18 Age: 50 (24–65)	Somatognatic treatment (SGT) comprising: occlusal splints, occlusal adjustments and exercise therapy	Not specified	VAS-intensity (0–100) NRS-severity (1–9)	Post-treatment, 6 months follow-up	**VAS-intensity:** Significant decrease after SGT or BFT (*n* = 31)
		vs.				
						No significant changes after SGT or BFT alone (*n* = 13 or 18)
	Design: RCT with cross-over design					
	Diagnosis: severe tinnitus and self-reported TMD or headaches	Biofeedback therapy (BFT) comprising biofeedback training, progressive relaxation and counseling				

### Risk of bias in the individual studies

The PEDro scale for randomized controlled trials was used to investigate the methodological quality of the included articles. This scale is recommended by the “Physiotherapy Evidence Database.” The PEDro scale was developed to rapidly identify clinical trials that are likely to be internally valid and have sufficient statistical information to make their results interpretable. The scale uses 11 items (Yes, No) to score each article on external validity (item 1), internal validity (item 2–9) and sufficient statistical information to make the results interpretable (item 10–11). A total score is calculated by summing the number of “Yes” answers on item 2–11. Item 1 is not taken into account for the total score.

The methodological quality assessment was performed by two investigators independently. Afterwards, the results were compared and differences were discussed to reach a consensus.

## Results

### Study selection

**Figure 1:**
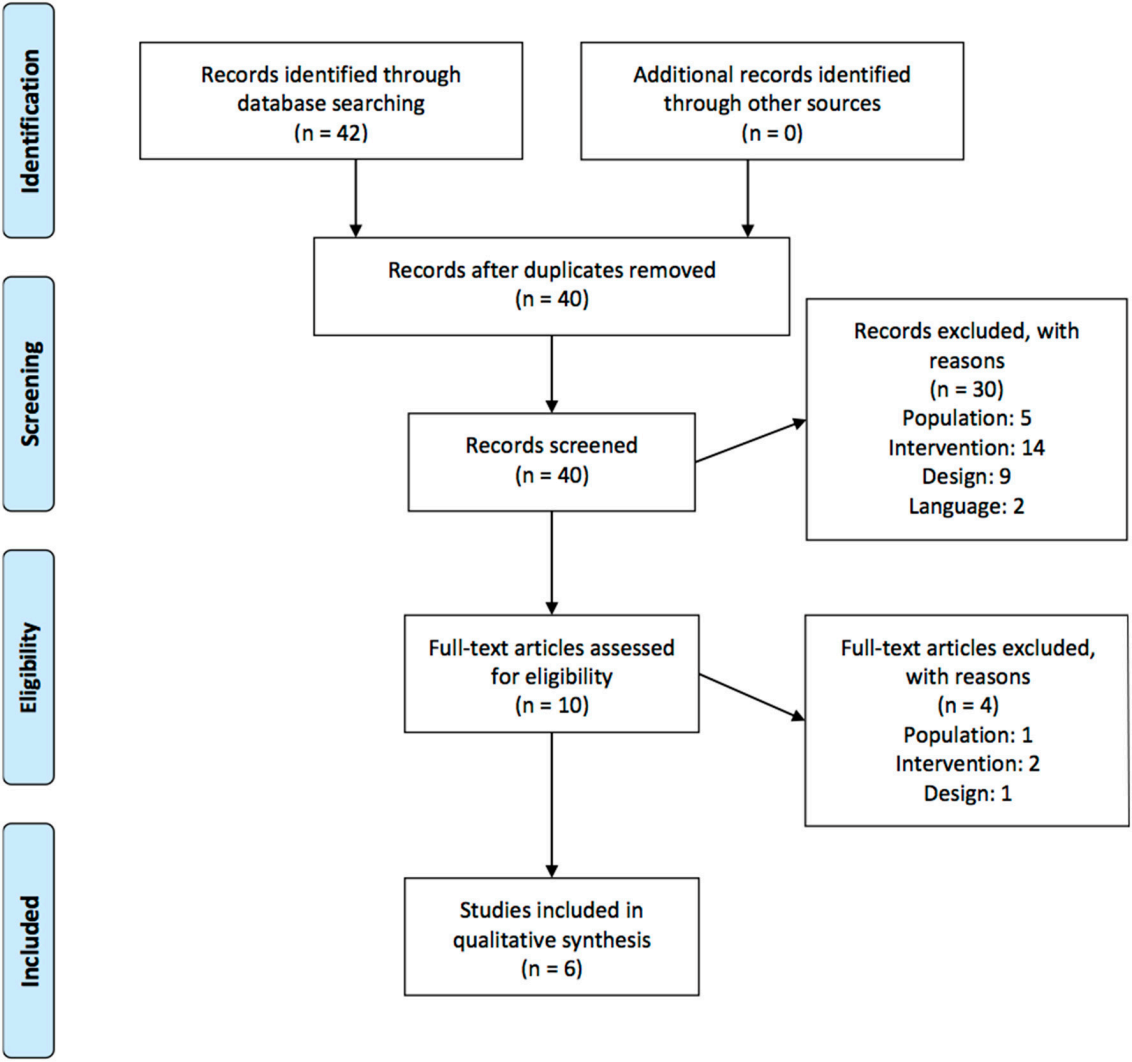
**Flowchart of study selection process**.

In total, 40 unique articles were retrieved from the 4 databases. After both screening phases, 6 articles were included in our review. In total, 6 studies were excluded due to the described population, 16 because the described intervention was not a physical therapy modality, 10 due to the design of the study (no comparison with “no treatment” or “another treatment” and 2 studies were excluded due to the language. A detailed overview of the selection process can be found in the flowchart in Figure 1.

### Risk of bias and level of evidence

**Figure 2:**
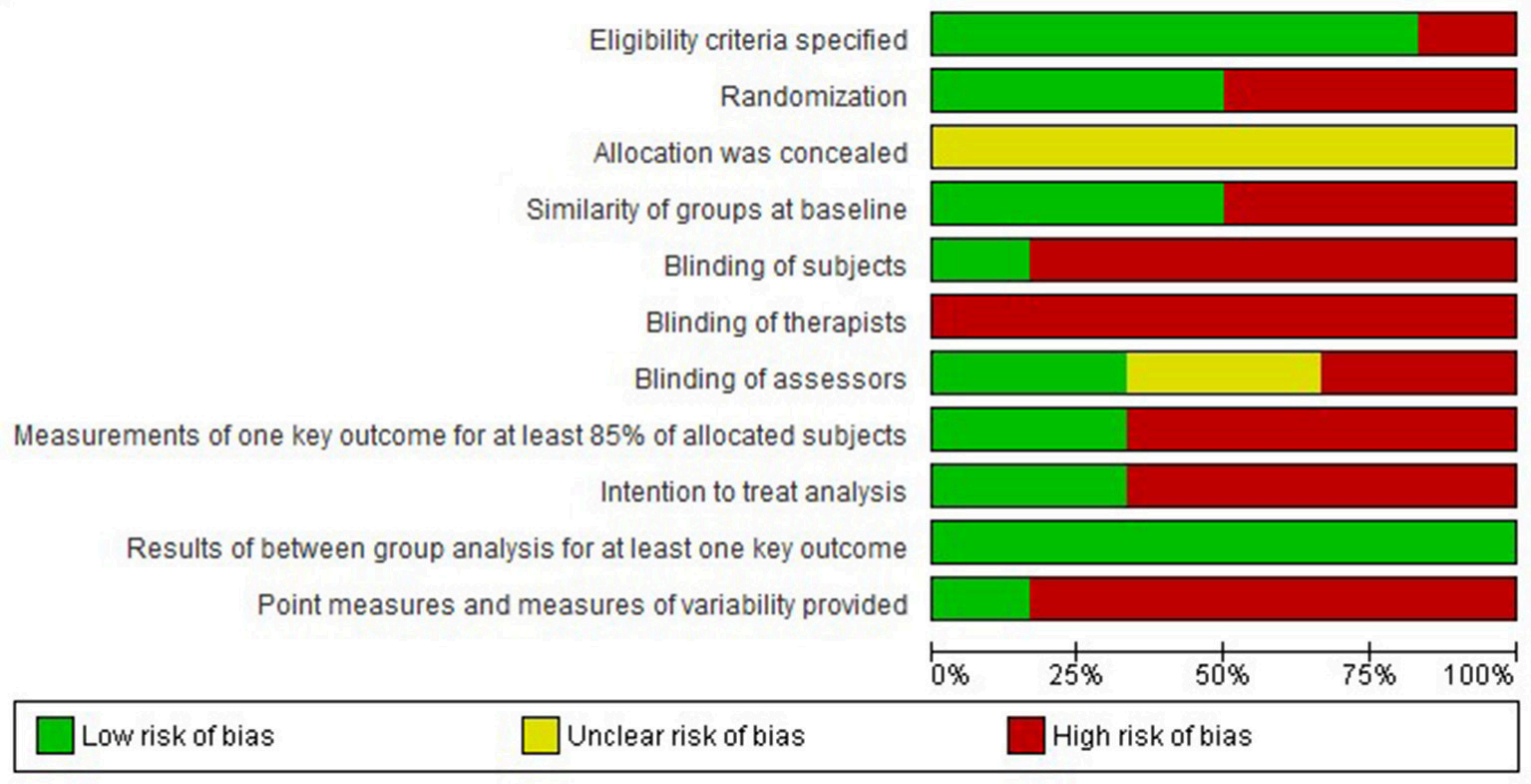
**Risk of bias graph: review authors' judgements about each risk of bias item presented as percentages across all included studies**.

**Figure 3:**
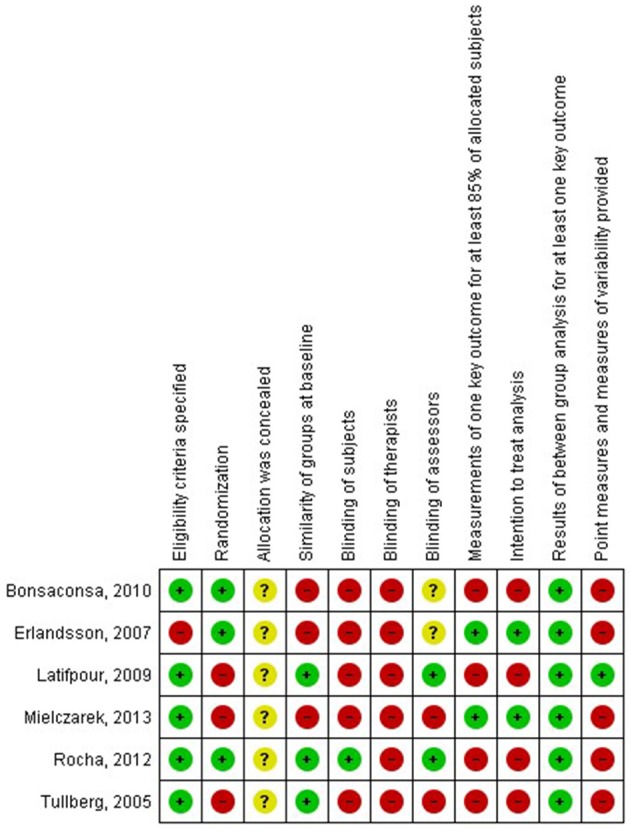
**Risk of bias summary: review authors' judgements about each risk of bias item for each included study**.

The results of the risk of bias assessment are presented in Figures 2, 3.

Overall, a high risk of bias was present in the included studies. This risk is mostly due to lack of randomization, lack of blinding of subjects, therapists, and/or investigators. Additionally, risk of bias is present due to incomplete presentation of the data and selective reporting. Therefore, the level of evidence of the included studies is low.

### Synthesis of the results

For each individual study, a summary of the characteristics of the study group, type of intervention and main results is presented in Tables 1, 2. Table 1 presents the studies concerning cervical spine treatment and Table 2 presents the studies regarding TMD treatment in tinnitus patients.

#### Cervical spine treatment

Four of the included studies investigated the effect of cervical spine treatment on tinnitus complaints. All of these studies had high risk of bias (Latifpour et al., 2009; Amanda et al., 2010; Rocha and Sanchez, 2012; Mielczarek et al., 2013), therefore the quality of the evidence is low.

Based on these studies, there are indications that cervical physical therapy (including stabilizing and mobilizing exercises) improves tinnitus complaints in a population of patients with a combination of tinnitus, sensorineural hearing loss and cervical spine degenerative changes (Mielczarek et al., 2013).

Additionally, there are indications that manipulations of the cervical spine decrease tinnitus severity (measured using the Tinnitus Handicap Inventory THI) in tinnitus patients. The intensity of the tinnitus (measured using visual analog scale VAS) did not decrease after cervical spine manipulations (Amanda et al., 2010).

Another study shows a significantly greater decrease in tinnitus severity (measured using VAS) after a combination of stretching, posture exercises and auricular acupuncture compared to waiting list (Latifpour et al., 2009) in patients with somatically related tinnitus.

Finally, there are indications that a combination of ischemic compression therapy of trigger points, stretching and posture exercises decreases tinnitus severity (measured using THI) in patients with tinnitus and pain complaints in head, neck, or shoulder girdle (Rocha and Sanchez, 2012).

#### Temporomandibular joint treatment

Two included studies investigated the effect of temporomandibular joint treatment on tinnitus complaints. Both studies had high risk of bias (Erlandsson et al., 1991; Tullberg and Ernberg, 2006), causing the quality of the evidence to be low.

TMJ treatment included splints, occlusal adjustments and jaw exercises in both studies. One study also added laser therapy (Tullberg and Ernberg, 2006).

Based on these studies, there are indications that TMJ treatment decreases tinnitus intensity (measured using VAS) and severity (measured using global perceived effect and a custom made questionnaire). TMJ treatment was more effective than no treatment and equally effective than a combination of biofeedback therapy, progressive relaxation and counseling.

## Discussion

The aim of this review was to investigate the current evidence regarding physical therapy treatment in patients with subjective tinnitus.

Physical therapy treatment was either directed to the cervical spine or the temporomandibular area. Overall, positive effects of physical therapy on tinnitus severity were found.

Regarding cervical spine treatment, the effect of exercise therapy on tinnitus severity was proven in two studies (Latifpour et al., 2009; Mielczarek et al., 2013) that treated 40 and 13 patients respectively. A positive effect of manipulations of the cervical spine was found in one randomized controlled trial (RCT) of 20 patients (Amanda et al., 2010) and improvement of tinnitus severity after combination of triggerpoint deactivation and exercise therapy was found in one study of 33 patients (Rocha and Sanchez, 2012).

Regarding TMJ treatment, the effectiveness of splints, occlusal adjustments and jaw exercises was shown in two studies (Erlandsson et al., 1991; Tullberg and Ernberg, 2006) of 104 patients in total.

Unfortunately, none of the data can be pooled, due to heterogeneity of inclusion criteria, outcome measures and applied treatment.

Firstly, an international standard of outcome measurements in clinical trials of tinnitus is lacking. This is mandatory to enable meta-analysis as was also pointed out by Hall et al. (2015). This international standard is being developed, but to date, a clear consensus was not reached yet.

Secondly, the lack of unambiguously composed diagnostic criteria for somatic tinnitus is reflected in the applied inclusion criteria. All researchers define their own inclusion criteria, making it very hard to compare studies and to pool data. Sanchez et al. (Sanchez and Rocha, 2011) suggested a series of diagnostic criteria in a literature review in 2011, but none of the studies used these criteria. Possibly due to the fact that the criteria, seem too broad, since somatic tinnitus is assumed in all patients where tinnitus and neck or TMJ complaints co-occur. Since a recent study (Michiels et al., 2015) showed that neck complaints also occur in patients with other types of tinnitus, modification of the diagnostic criteria for somatic tinnitus is needed. Additionally, when applying cervical spine or TMJ treatment, studies should only include those patients that require this therapy for the treatment of their neck or TMJ complaint. In the study of Amanda et al. (2010) for instance, patients were included in case they had tinnitus and were otherwise healthy. These patients were treated using manipulations of the cervical spine, a treatment modality that is normally performed in case of limited range of motion of the cervical spine. The presence of these limitations in range of motion were however, not a requirement for patients to be included in the study. Therefore, doubts about the usefulness of manipulations in the study population may arise and therapy effects may be underestimated.

Another study (Mielczarek et al., 2013) included patients based on the presence of radiologically confirmed degenerative changes in the cervical spine, though degeneration is not necessarily accompanied with dysfunction and cervical spine complaints.

Thirdly, the applied treatments for cervical spine and TMJ complaints are divergent and do not always match the evidence based practice for cervical spine or TMJ treatment. In patients with somatic tinnitus, tinnitus severity is thought to be altered by cervical spine or TMJ dysfunctions. Therefore, complete alleviation of cervical spine or TMJ complaints using the best available treatment option will be necessary for a maximal decrease in tinnitus severity. Systematic reviews (Kay et al., 2005; Gross et al., 2010, 2015; Miller et al., 2010; Schroeder et al., 2013) have shown that a multimodal physical therapy treatment, combining mobilizations/manipulations and exercises is the best treatment option for cervical spine complaints. For TMJ complaints, treatment options vary (not only in specific choices for exercise or mobilization, but also the care provider), depending on the diagnosis and etiology. Both dentists and physical therapists may play a role in the primary care treatment of these patients (Feine and Lund, 1997; de Souza et al., 2012). Future studies should include these approaches to investigate its effect on tinnitus severity in patients with somatic tinnitus. The use of evidence based cervical spine and TMJ treatment instead of less underpinned therapies is specifically important in patients who already received numerous unsuccessful therapies in the past, as is the case in many patients with tinnitus.

All six studies however, show high risk of bias, limiting the generalizability of the conclusions. The risk was mostly due to lack of randomization, lack of blinding of subjects, therapists and/or assessors, and additionally due to incomplete presentation of the data and selective reporting. Lack of randomization was mostly caused by practical considerations, such as decreasing the waiting period before the start of the treatment. Blinding of subjects and therapists is always an issue in studies investigating physical therapy treatment and is very hard to overcome. Therefore, blinding of the assessor, who performs the follow-up measurements and data processing, is even more important. Although blinding the assessor is perfectly possible in physical therapy studies, only two studies mentioned this type of blinding (Rocha and Sanchez, 2007; Latifpour et al., 2009). Selective reporting and incomplete data presentation was another issue in the included articles. Only two out of six articles (Erlandsson et al., 1991; Mielczarek et al., 2013) presented the results of measurements of at least 85% of the allocated subjects. Additionally, only one study (Latifpour et al., 2009) provided point measures and measures of variability, where most other studies only provided significance figures. These issues of lack of randomization and blinding of assessors and selective reporting should be avoided in future research.

In future studies, researchers should firstly focus on clear patient selection, based on the existing diagnostic criteria and the applied treatment. Secondly, evidence based cervical spine and TMJ treatments should be applied and thirdly, studies should prevent risk of bias by focusing on randomization, blinding of assessors, and complete reporting of data.

## Conclusion

Despite the methodological issues in the included studies and the consequent low quality evidence, it is noteworthy that all included studies showed positive treatment effects. Although the results of the 6 studies are promising, the quality of the studies do not reach a high EBM level, which is necessary to endorse clinical practice and experience with recommendations. Current available effectiveness methodology and assessment has to guide future studies. These studies should focus on larger populations, higher methodological quality and should use unambiguous inclusion criteria, state-of-the-art treatment, and high quality outcome measures.

## Author contributions

The literature was screened and methodological quality was assessed independently by SM and SN. WD supervised the process. PV and AG provided overall expertise on tinnitus complaints and MB and CV provided overall expertise on temporomandibular dysfunction. SM drafted the manuscript and all other authors added their comments.

### Conflict of interest statement

The authors declare that the research was conducted in the absence of any commercial or financial relationships that could be construed as a potential conflict of interest.
